# Role of Numb expression and nuclear translocation in endometrial cancer

**DOI:** 10.3892/ol.2015.2901

**Published:** 2015-01-27

**Authors:** CHAO WANG, TAO CUI, WEIWEI FENG, HUASHUN LI, LINA HU

**Affiliations:** 1 Department of Obstetrics and Gynecology, Hospital of Fudan University, Shanghai, P.R. China; 2 Department of Obstetrics and Gynecology, West China Second Hospital, Sichuan University, Chengdu, Sichuan, P.R. China; 3 Developmental and Stem Cell Institute, West China Second Hospital, Sichuan University, Chengdu, Sichuan, P.R. China; 4 Department of Obstetrics and Gynecology, Second Affiliated Hospital of Chongqing Medical University, Chongqing, P.R. China

**Keywords:** Numb protein, endometrium cancer, nuclear translocation

## Abstract

The cell fate determinant Numb orchestrates numerous cell physiological and pathological processes and previous evidence has indicated that Numb expression is associated with tumorigenesis. The present study investigated the potential role of Numb in endometrial cancer (EC). Numb expression was compared between the normal endometrium and EC tissue by immunohistochemistry, and the protein levels were assessed by western blotting and confocal microscopy in the human endometrial HEC-1B cancer cell line and normal epithelial cells. The intracellular localization of Numb in HEC-1B cells was examined by immunofluorescence. Numb was found to be expressed at higher levels in endometrial cancer compared with the normal endometrium. Although Numb localizes to the cytoplasm and plasma membrane in the normal epithelium, the present study found that Numb accumulated in the nucleus of HEC-1B cells. The present data reveals the upregulation of Numb expression in EC tissues and indicates that Numb plays a role in the occurrence of EC, which may be mediated by its translocation into the nucleus. The role of Numb in cancer development requires additional investigation.

## Introduction

Endometrial cancer (EC) is one of the most common gynecological malignancies that affect the health of women worldwide (1). An increase in the incidence and morbidity of EC in young women, in whom the preservation of fertility is of particular importance, indicates the importance of identifying treatment options for this disease, as the current standard treatment for EC is total hysterectomy (2). Therefore, the pathogenetic mechanism of EC has been the subject of extensive research efforts in previous decades. The tumorigenesis of EC is a complicated process that involves multiple factors, stages and gene mutations. Numerous factors or processes involved in the pathogenesis of EC remain unidentified, although several factors closely associated with the occurrence of EC are known, including oncogenes, anti-oncogenes, estrogen, progestin, estrogen and progestin receptors, DNA mismatch repair genes and satellite instability (3,4).

Numb is an evolutionarily conserved developmental protein that plays a critical role in cell-fate determination and differentiation. Numb was first identified as a mediator of cell division in neural progenitors in Drosophila, in which asymmetric segregation of Numb results in the two daughter cells acquiring different fates (5). Previous research has revealed an association between Numb expression and cancer development (6–11) that involves several important cellular processes, including cell polarity, cell division and epithelial to mesenchymal transition (EMT), as well as multiple signaling pathways, such as Notch, Hedgehog and p53 (8). In addition, numerous proteins have been identified as binding partners for Numb, including the Par3-Par6-aPKC complex, E-cadherin, integrin, ligand of Numb-protein X and the dualoxidase activator/Numb interacting protein (12–15). As these pathways and proteins are involved in the onset and metastasis of several malignancies, it is reasonable to assume that Numb plays a role in cancer development.

The role of Numb as a tumor suppressor in breast cancer has been proposed by several studies that reported that the removal of Numb resulted in reduced TP53 levels and impaired TP53, apoptosis and DNA-damage checkpoint activation. In addition, the tumor suppressor role of Numb was demonstrated to be associated with the formation of the Numb/TP53/MDM2 complex (16). Reduced Numb expression was correlated with decreased disease-free survival and a higher risk of developing distant metastases from breast cancer (17). However, the role of Numb in tumorigenesis differs in other types of tumors. In experimental glioma, Numb overexpression does not exert a tumor suppressor function and does not impair cell proliferation *in vitro* or induce differentiation of neural or glial cells (18). Additionally, two newly-identified isoforms of Numb, Numb5 and Numb6, have been revealed to act as oncogenes (9).

Regardless of its exact function, the Numb protein has been increasingly associated with tumorigenesis. However, there are no reports on the role of Numb in EC. In the present study, the potential association between Numb and EC was investigated and, to the best of our knowledge, the present study demonstrated for the first time that Numb plays a role in the development of EC.

## Materials and methods

### Cell culture and clinical samples

Human embryonic kidney 293T cells were obtained from the Developmental and Stem Cell Institute of West China Second University Hospital (Chengdu, Sichuan, China). Human endometrial HEC-1B cancer cells were obtained from the Gynecological Oncology Laboratory of West China Second University Hospital. The cell lines were cultured in Dulbecco’s modified Eagle’s medium-high glucose (DMEM-HG; Gibco Life Technologies, Carlsbad, CA, USA) supplemented with 10% fetal bovine serum (Hyclone, Logan, UT, USA), 40,000 mU/ml penicillin and 40 μg/ml streptomycin (both Corning Life Sciences - Mediatech Inc., Manassas, VA, USA), at 37°C in a humidified incubator with a 5% CO_2_ atmosphere. Upon reaching 90% confluence, the cells were dissociated using 0.25% trypsin and subcultured.

Between August 2008 and March 2009, eight patients with EC, aged between 37 and 81 years, who had been treated at the Department of Gynecology and Obstetrics (West China Second University Hospital), were enrolled in the present study. Hysterectomy, bilateral salpingo-oophorectomy, lymphadenectomy and cytological examination of the peritoneal fluid were performed. Patients who were not found to possess macroscopic lesions during the procedure and who received a non-EC post-operative pathological diagnosis, such as cervical cancer, were excluded. For each patient, normal endometrial and EC tissues were collected and promptly stored in 4% paraformaldehyde (PFA) subsequent to being obtained. Patients lacking a normal endometrium were excluded. The present study was approved by the Medicine Ethics Committee of West China Second Hospital of Sichuan University. Consent was obtained from the patients prior to collecting the patient-derived tissues.

### Immunohistochemistry

The tissue was fixed in 4% PFA and then embedded in paraffin. A standard immunohistochemical staining procedure was performed. Briefly, a deparaffinization series was performed using xylene and ethanol. Antigen retrieval was achieved by boiling tissue slides with 0.01 mol/l citric buffer. Hydrogen peroxide was used to quench the endogenous peroxidase activity. Subsequent to blocking, the sections were incubated with a polyclonal rabbit anti-human Numb72 antibody (1:1,000; Developmental and Stem Cell Institute of West China Second University Hospital) overnight at 4°C. The sections were incubated with the corresponding biotinylated goat anti-rabbit immunoglobulin (Ig)G secondary polyclonal antibodies (catalog number, 111-065-003; Jackson Immunoresearch, West Grove, PA, USA) for 1 h at room temperature followed by incubation with peroxidase-conjugated streptavidin (catalog number, 016-030-084; Jackson Immunoresearch) for 30 min at room temperature and then were stained with 3,3′-Diaminobenzidine. The stained slides were counterstained with hematoxylin, dehydrated using alcohol and xylene and mounted in resinous mounting media. The tissue sections stained with isotype IgG were used as controls. All the slides stained with the same antibody were processed simultaneously. The stained tissue slides were analyzed under an Olympus CKX41 microscope (Tokyo, Japan), and images were captured by a digital camera DP70, Olympus) and recorded into a microscope-linked PC computer. Average intensity quantification was performed using Image-Pro Plus version 6.0 software (Media Cybernetics, Inc., Rockville, MD, USA). Intensity was measured in three equally divided regions. Average intensity per area was determined by dividing the sum of all pixel intensities by the measured area. All compared images were acquired under identical parameters. The data were expressed as the mean ± standard error of the mean.

### Immunofluorescence (IF) analysis

Cells growing on coverslips were fixed with 4% PFA for 15 min at room temperature and then washed three times with PBS. Subsequent to blocking for 30 min, the coverslips were incubated with a polyclonal rabbit anti-human Numb72 antibody (Developmental and Stem Cell Institute of West China Second University Hospital) and mouse anti-human nuclear pore complex (NPC) proteins monoclonal antibody (1:1,000; catalog number, MMS-120R; Covance, Inc., Princeton, NJ, USA) overnight at 4°C. The cells were incubated with the corresponding polyclonal cy2 anti-rabbit (green fluorescence; 1:2,000; catalog number, 111-225-003) and polyclonal cy3 anti-mouse (red fluorescence; 1:2,000; catalog number, 111-165-003) secondary antibodies (Jackson Immunoresearch) for 1 h at room temperature in the dark, and the coverslips were mounted in antifade mounting media. The cells were observed with a confocal laser microscope (LSM510; Zeiss, Oberkochen, Germany). Images were captured using the LSM510 microscope, and fluorescence intensity quantification was performed by Image-Pro Plus version 6.0 software. Fluorescence intensity was measured in six equally divided regions. Average fluorescence intensity per area was determined by dividing the sum of all pixel intensities by the measured area. All compared images were acquired under identical parameters. The data were expressed as the mean and standard error of the mean.

### Immunoblots

Lysates were extracted from five cell lines and homogenized in lysis buffer [50 mM Tris-HCl, pH7.4; 1% NP-40; 0.25% sodium deoxycholate; 150 mM NaCl; 1 mM EDTA; and 1 mM PMSF, supplemented with 1 μg/ml protein inhibitor cocktail (P2714, Sigma-Aldrich, St. Louis, MO, USA) (prior to use)]. The protein concentrations were determined by the bicinchoninic acid protein assay. Equal amounts of protein were separated on a 10% SDS polyacrylamide gel and transferred to PVDF membranes. Subsequent to blocking for 1 h, the membranes were incubated with monoclonal primary rabbit anti-human Numb (1:1,000; catalog number, 2756; Cell Signaling Technology, Inc., Danvers, MA, USA) and monoclonal mouse anti-human β-actin (1:2,000; catalog number, sc47778; Santa Cruz Biotechnology, Inc., Dallas, TX, USA) antibodies overnight at 4°C in blocking buffer, which consisted of Tris-buffered saline containing 5% skim milk and 0.1% Tween-20 (TBS-T). The primary antibody-bound membranes were washed three times in TBS-T and incubated for 1 h at room temperature with horseradish peroxidase-conjugated secondary polyclonal goat anti-rabbit IgG antibody (1:3,000; catalog number, ZB2301; Zhongshan Golden Bridge, Beijing, China) and polyclonal goat anti-mouse IgG antibody (1:3,000; catalog number, ZB2305; Zhongshan Golden Bridge, Beijing, China). Immunosignals were visualized using the Immun-Star WesternC chemiluminescence kit (product number, 170–5070; Bio-Rad Laboratories, Hercules, CA, USA). Each experiment was repeated three times. Intensity determination was performed using Image-Pro Plus 6.0 software (Media Cybernetics, Inc., Rockville, MD, USA).

### Statistics

All statistical tests were performed using SPSS Statistics, version 13.0 (SPSS, Inc., Chicago, IL, USA). P<0.05 was considered to indicate a statistically significant difference.

## Results

### Numb expression in normal endometrial and EC tissues

**Figure 1:**
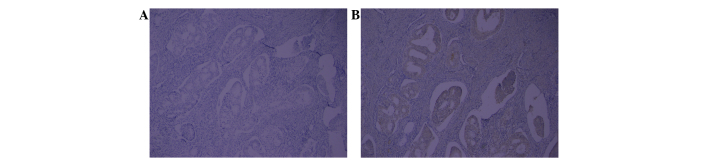
Numb72 expression in endometrial cancer (EC). The expression of Numb72 was analyzed by immunohistochemistry in EC tissue. The brown color indicates Numb72 expression. (A) Negative control (magnification, ×10). (B) Positive expression of Numb72 (magnification, ×10).

The expression of Numb72, one of four Numb isoforms, was analyzed by immunohistochemistry (IHC) in the tissues of five EC patients selected at random, and expression of the protein was detected in all cases (Fig. 1).

**Figure 2:**
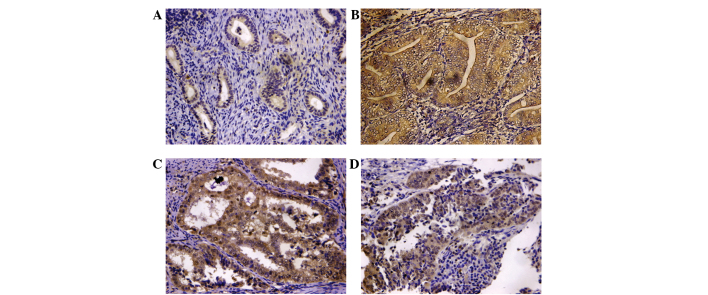
Numb72 expression in normal endometrial and endometrial cancer (EC) tissues. Numb72 expression was compared between EC and normal endometrial tissues. The brown color indicates Numb72 expression (magnification, ×40). (A) Normal endometrial tissue. (B) High-medium grade EC tissue. (C) Medium grade EC tissue. (D) Low grade EC tissue.

**Figure 3:**
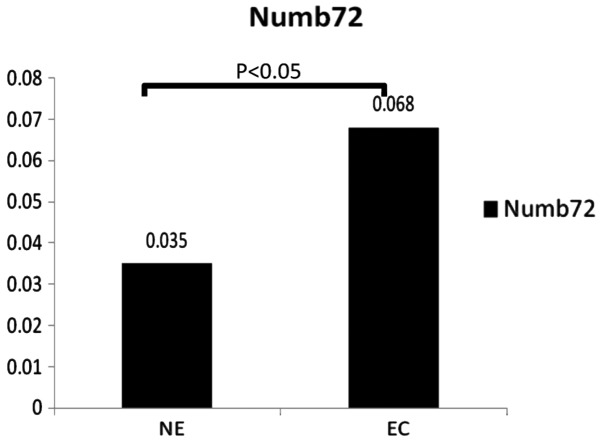
Quantification of the immunohistochemical staining for Numb72 expression in NE and EC tissues. Numb72 expression in the EC tissue was 0.068±0.036 compared with 0.035±0.01 in the NE (P<0.05). NE, normal endometrium; EC, endometrial cancer.

The pathological characteristics of eight patients with EC are described in Table I. IHC analysis revealed that Numb72 was expressed at higher levels in EC tissues compared with normal endometrial tissue (0.068±0.036 vs. 0.035±0.01, respectively; P<0.05; Figs. 2 and 3). In addition, the brown particles demonstrating Numb72 expression were detected with a higher frequency in the nucleus of EC tissues (Fig. 2).

### Numb72 expression in HEC-1B and 293T cells

#### IF detection of Numb72

**Figure 4:**
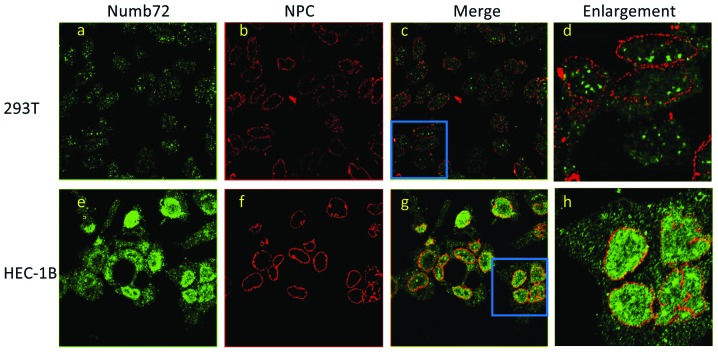
Numb72 expression in 293T and HEC-1B cells. Immunofluorescence was used to examine the expression level and intracellular localization of Numb in 293T and HEC-1B cells. Green indicates Numb72 expression, red indicates the nuclear pore complex (NPC). (a) Numb72 expression in 293T cells. (b) NPC in 293T cells. (c) Expression of Numb72 and the NPC in 293T cells. (d) Magnification of the blue frame in (c). (e) Numb72 expression in HEC-1B cells. (f) NPC in HEC-1B cells. (g) Expression of Numb72 and the NPC in HEC-1B cells. (h) Magnification of the blue frame in (g). Magnification for parts (a-c) and (e-g), ×63.

**Figure 5:**
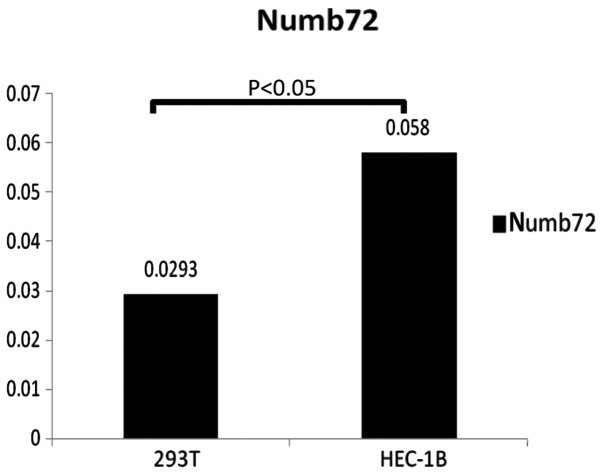
Quantification of immunofluorescent staining for Numb72 expression in 293T and HEC-1B cells. The experiment was repeated three times. Numb is expressed at higher levels in HEC-1B compared with 293T cells (0.058±0.004 vs. 0.0293±0.018; P<0.05).

IF and laser scanning confocal microscopy (LSCM) were used to study the intracellular localization and expression of Numb in the 293T and HEC-1B cell lines. The results revealed that Numb predominantly localized to the cytoplasm in 293T cells and the nucleus in HEC-1B cells, and that Numb72 expression levels were higher in HEC-1B cells compared with 293T cells (0.058±0.004 vs. 0.0293±0.018; P<0.05; Figs. 4 and 5). The cells were probed with an antibody against NPC proteins that specifically detects the location of the nuclear membrane to identify the location of Numb, which confirmed the aforementioned results.

#### Western-blot analysis of Numb72

**Figure 6:**
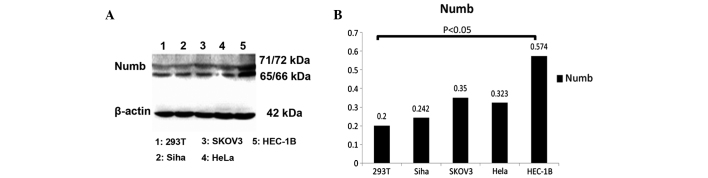
Numb72 protein levels in 293T and HEC-1B cells. Western blotting was used to determine the expression level of the Numb protein in different cells. The experiments were repeated three times. (A) Western blot analysis results. (B) Quantification of the western blotting results for Numb72 expression in 293T and HEC-1B cells revealed that the highest level was present in the HEC-1B cells, with a statistically significant difference between HEC-1B and 293T cells (0.574±0.17 and 0.198±0.08, respectively; P<0.05).

Numb72 expression was analyzed by western blotting in the Siha, SKOV-3, HeLa, HEC-1B and 293T cell lines to determine whether the levels of the Numb72 protein differ between normal cells and cancer cells. The results revealed that Numb expression is highest in HEC-1B cells, with a statistically significant difference in the levels of the protein between HEC-1B and 293T cells (0.574±0.17 and 0.198±0.08, respectively; P<0.05; Fig. 6).

## Discussion

Mammalian Numb encodes four alternatively spliced transcripts that generate four proteins ranging between 65 and 72 kDa in size. Numb contains an amino-terminal phosphotyrosine-binding domain (PTB) and C-terminal proline-rich region (PRR), comprising putative Src homology 3-binding sites, and an Eps15 homology (EH) region [DPF; responsible for binding to alpha-adaptin, and NPF; responsible for binding to the EH domain of endocytic proteins). The various domains of Numb possess different functions. The PTB domain is crucial for Numb function as PTB domains are protein interaction domains, whereas the DPF and NPF motifs are critical for the role of Numb as an endocytic adaptor protein. A large 48-amino acid insert in the PRR region distinguishes the Numb1 and Numb3 isoforms from Numb2 and Numb4, which lack this sequence. A smaller 11-amino acid insert in the PTB region also distinguishes Numb1 and Numb2 from Numb3 and Numb4 (19). In the present study, Numb72 was selected since it has been associated with the proliferation and differentiation of cells, suggesting that Numb may be a candidate factor involved in the association between Numb proteins and cancer. Numb72 localizes to the plasma membrane due to the insertion of 11 amino acids in the PTB region (19–21).

The present results revealed that Numb72 expression was higher in EC compared with normal endometrial tissue, and Numb72 localized to the plasma membrane and the nucleus in EC, which was consistent with the results obtained from cervical cancer cells (22). In the present study, the asymmetric distribution of Numb in the apical membrane of cells was not observed as has been reported in neurons, in which Numb segregates to the apical daughter cell that remains as a progenitor. Although the reasons for this discrepancy are not clear, it may be due to differences in the experimental methods used or the analysis of the protein at various cell cycle phases, as Numb was demonstrated to localize to the plasma membrane during the mitosis phase, while during interphase, Numb is phosphorylated and distributed into the cytoplasm (23). In addition, the present results revealed that Numb expression was gradually upregulated in correlation with the differentiation grade of EC tissue. The intracellular localization of Numb may be associated with tumorigenesis and the degree of malignancy of tumors, although further research with a larger number of clinical samples is necessary to reach a statistically significant conclusion.

The expression of Numb in HEC-1B and 293T cells was analyzed by LSCM, which not only confirmed the upregulation of Numb72 in HEC-1B cells compared with 293T cells, but also revealed the predominant nuclear localization in HEC-1B cells. The nuclear localization of Numb72 in HEC-1B cells was confirmed by the co-localization of Numb72 with NPC proteins in the nucleus. The present immunohistochemical and IF results clearly indicate that Numb72 expression is not restricted to the cytoplasm and plasma membrane as previously reported, but that the protein is also expressed in the nucleus of EC cells. This may indicate a translocation of the overexpressed Numb72 protein from the cytoplasm to the nucleus in EC cells. This result was confirmed in cells overexpressing Numb72 following transfection with the pD-RFP-numb72 plasmid (data not shown). The present data suggest that increased expression of Numb72 may be associated with tumorigenesis and its nuclear translocation may be a key underlying mechanism.

Overall, the present results suggest that Numb may be involved in the pathogenesis of EC. Furthermore, Numb does not appear to play a protective role in EC, and its nuclear translocation may represent a novel pathogenetic mechanism of EC development. As Numb inhibits Notch signaling in the cytoplasm, the translocation of Numb from the membrane or cytoplasm into the nucleus may activate Notch signaling (24,25). The role of Numb in the regulation of p53 activity is not clear. Colaluca *et al* reported that Numb protects p53 from MDM2-mediated degradation, and decreased levels of Numb result in the downregulation of p53, leading to the occurrence of breast cancer (16). This study also reported that Numb forms a tricomplex with p53 and MDM2. However, in the present study, increased Numb expression and nuclear translocation in EC cells was correlated with increased p53 expression in the nucleus (data not shown), suggesting that Numb may act in association with p53 and MDM2 in the nucleus of endometrial cancer cells in a different manner than in breast cancer cells. The role of Numb in tumorigenesis should be explored in further detail in future studies.

## Figures and Tables

**Table I:** The pathological characterises of eight patients with endometrial cancer.

Patient	Age, years	Histological subtype	Stage	Depth of myometrium invasion	Vascular metastasis	Lymph node invasion
1	49	Medium-low grade endometroid EC	Ic	Whole	No	No
2	81	Low grade endometroid EC	Ia	No	No	No
3	50	Medium grade endometroid EC	Ib	<1/2	No	No
4	58	Medium-low grade endometroid EC	Ia	No	No	No
5	51	Low grade endometroid EC	Ib	<1/2	No	No
6	59	Low grade endometroid EC	Ib	<1/2	Yes	No
7	37	High-medium grade endometroid EC	Ib	<1/2	No	No
8	56	Medium-low grade endometroid EC	Ib	<1/2	No	No

EC, endometrial cancer.
